# Research on routing and scheduling algorithms for the simultaneous transmission of diverse data streaming services on the industrial internet

**DOI:** 10.1038/s41598-021-97613-9

**Published:** 2021-09-15

**Authors:** Yan Song, Chenyang Guo, Panfeng Xu, Lina Li, Rui Zhang

**Affiliations:** 1 College of Physics, Liaoning University, Shenyang, 110000 China; 2 Shenyang Institute of Automation, Chinese Academy of Sciences, Shenyang, 110000 China

**Keywords:** Computer science, Information technology

## Abstract

OPC UA PubSub Over TSN is the core of the Industrial Internet and guarantees flexible interaction features for multiple parties in real-time for industrial communication. To achieve the transmission of time-triggered traffic in PubSub NetworkMessage, routing and scheduling data need to be analyzed. Traditional routing and scheduling methods have disadvantages such as low calculation efficiency, slow convergence speed, and poor reliability. Therefore, a routing and scheduling method for OPC UA PubSub NetworkMessage time-triggered traffic based on an improved ant colony algorithm is proposed. First, we analyze the network topology model, traffic model, and traffic transmission constraints of TSN; then, we apply the K-means clustering algorithm, the KSP algorithm based on the shortest path idea, and an improved ant colony algorithm for traffic classification, routing, and scheduling calculation. Experimental results show that this method can effectively reduce the delay increase caused by link congestion, improve the ability to schedule time-triggered traffic, and accelerate the convergence rate of iteration.

## Introduction

With the development of the Industrial Internet^1^, the integration of information technology (IT) and industrial technology (OT) has been greatly accelerated^2^. Smart Factory, a product of the Industrial Internet, promotes the integration of heterogeneous networks with a distributed control system, making simultaneous transmission of data streaming services a reality, including enterprise resource planning (ERP) data, product lifecycle management (PLM) data, factory and workshop manufacturing execution system (MES) data, and office data. In addition, in the transmission of diverse data streaming services, the integration of OPC UA and TSN technologies can effectively ensure the real-time and semantic interoperability of data streams.

### OPC UA

**Figure 1:**
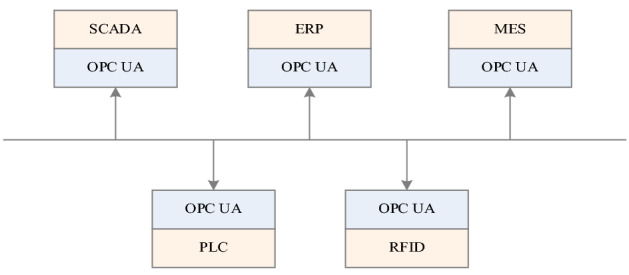
Integration of OPC UA in the automation.

OLE for Process Control Unified Architecture (OPC UA)^3^, is the standard meeting platform for industry integration and information sharing. It virtualizes physical devices into an OPC UA address space containing multi-sided service architecture (SOA) tags^50^. It integrates data access, alarm information, event information, and historical data into one accessible address space^4^. Each tag contains real-time control data, project specifications, and equipment information. By standardizing the format of data exchange, real-time control, access to the physical equipment can be achieved^1,5^. Access to different devices is designed to ensure interoperability and data integration, ensuring that physical devices communicate seamlessly with the network. In addition, OPC UA has advanced self-organization capabilities. The manufacturing system not only realizes the horizontal integration of access to different devices (PLC, RFID, etc.), but also the vertical integration of SCADA, ERP, and MES data. This transforms the manufacturing system into a decentralized organization^5–7^, as shown in Fig. 1.

### Time sensitive network

The main purpose of the IEEE802.1 time sensitive network (TSN) is to service time-sensitive projects and systems^8^. According to the requirements of different data streams for quality of service (QoS), it transmits a variety of traffic on the same Ethernet link, including Time-Triggered traffic, Audio–Video Bridge traffic, and Best-Effort traffic. Clock synchronization and transmission scheduling enable data to be transmitted with the smallest possible delay and jitter to facilitate the coexistence of BE, TT, and AVB traffic^8–10^. At present, commonly used protocols include: IEEE802.1 AS^11^, IEEE802.1 Qat^12^, IEEE802.1 Qav^13^, IEEE802.1 Qbu^14^, IEEE802.1 Qbv^15^ and IEEE802.1 Qcc^16^.

### OPC UA over TSN

With the current advancement of the IoT and Industry 4.0, automation manufacturers are eager to evolve from the automation model to the “self-hosted character tower model”, combining OPC UA with TSN^17,18^. As the standard application-layer interface for automated manufacturing hardware, OPC UA integrates address space and provides semantic operations to achieve OPC UA server access to data on hardware devices^19^. Furthermore, users can add a TSN tag to the head of the OPC UA data stream to enable it to be transmitted throughout the TSN network. To ensure certainty and reliability, vertical integration from the application layer to the data link layer is created^20^.

### Work of this paper

In this paper, we propose a method to directly map OPC UA NetworkMessage data from the application layer to the TSN network. To determine the TT traffic of NetworkMessage data, we apply a clustering algorithm to classify NetworkMessage information in a real-time network data set. In addition, we use the BA model to generate a static network topology and combine it with the KSP algorithm to reduce the routing search space. Finally, we thoroughly consider the impact of the transmission constraints of TT traffic on global scheduling performance and apply the improved ant colony algorithm to optimize TT traffic scheduling. Our algorithm converges faster than other available methods and best solves the routing and scheduling problems of OPC UA NetworkMessage on TSN networks.

## Related work

In research of OPC UA Over TSN, Bucknerder et al.^21^, Tian and Hu^22^ developed cross-level communication of the automatic font tower through the establishment of the OPC UA Over TSN model, making network communication horizontal and simplifying the network transmission process. Kobzan et al.^23^ proposed a TSN network for OPC UA based on SDN configuration and designed and verified the IEEE802.1 Qbv standard. Andreas Eckhardt and Sebastian Müller developed the round-trip time test for a point-to-point communication process based on a development board, and integrated the two standards of IEEE802.1 Qbv and IEEE802.1AS.

In research of TSN frames routing and scheduling, Pahlevan and Obermaisser proposed a genetic algorithm-based heuristic scheduling method, which improves the efficiency of scheduling, transmission, and link utilization for TT traffic^24^. Sune and Steiner proposed search space reduction technology and a heuristic algorithm based on a greedy randomized adaptive search process to inform AVB traffic of the worst-case end-to-end delay and minimal network utilization^25^. Combining the TT Ethernet protocol, Steiner et al. proposed a meta-heuristic method based on Tabu Search that achieves comprehensive TT traffic and RC traffic scheduling and provides minimal end-to-end delay for RC traffic^26^. Bingqian and Yong et al. proposed a Hybrid-GA-based time-triggered Ethernet static scheduling algorithm, which effectively increases the speed of the scheduling process and improves scheduling flexibility of the TT Ethernet^27^. Wang et al. proposed an improved ant colony algorithm for routing and scheduling of time-triggered traffic, which provides deterministic network delay and jitter for time-triggered traffic transmission^28^.

## OPC UA over TSN mapping and system model

### OPC UA over TSN mapping

OPC UA and TSN are standards for the application layer and data link layer, respectively. The combination of OPC UA and TSN is one of the cores of the Industrial Internet, providing real-time and interoperability for data streams. To ensure that OPC UA NetworkMessage data can be transmitted over the TSN network, the NetworkMessage data must be mapped to the TSN frame. OPC UA provides two communication modes, CS mode and PubSub mode. PubSub mode provides multi-to-multi communication and periodic data transfer between devices. Because the transmission of NetworkMessage data to the TSN network is periodic, we perform the mapping from OPC UA to TSN through PubSub mode^29^.Compared with XML and JSON, binary-coded PubSub NetworkMessages are adapted to the transmission environment with high frequency and low bandwidth occupation^29,30^. To provide a security mechanism for data transmission, we send data in a binary-coded mode.

**Figure 2:**
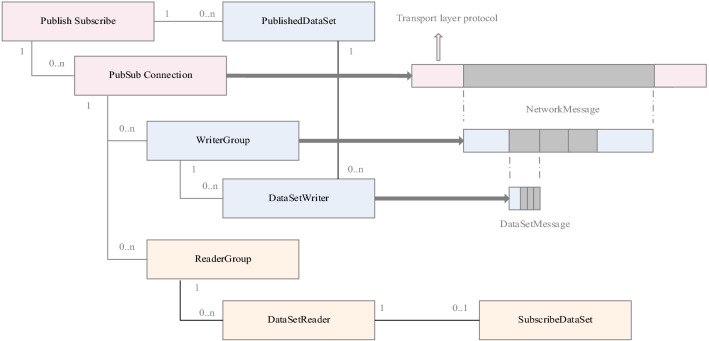
OPC UA PubSub configuration.

As shown in Fig. 2, the specific mapping process of NetworkMessage data to the TSN data stream is as follows.

**Figure 3:**
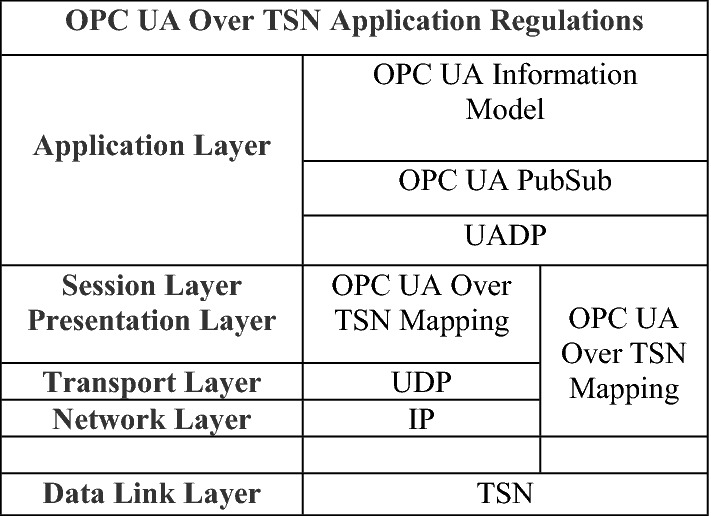
OPC UA over TSN for converged architecture protocol.

Through configuring OPC UA PubSub, a concrete example was created. PublishSubscrible is the root node of the object information model and contains the data content of PubSub. A NetworkMessage is the object of a WriterGroup. The WriterGroup contains at least one DataSetWriter sub-object, and the DataSet encapsulates the DataSetMessage in the DataSetWriter object and the PublishedDataSet binding. The PublishedDataSet contains metadata and encoding methods that describe the data set. The NetworkMessage is the final payload of the application layer, transmitted to the bottom layer, and finally completing the mapping as the TSN data frame's payload. The mapping from the application layer to the data link layer is divided into two forms, as shown in Fig. 3.

1. Application layer NetworkMessage passes through the transport layer and network layer, and finally maps to the data link layer;
2. Application layer NetworkMessage directly maps to the data link layer;

The message of form (1) needs to be encoded by the UDP at the transport layer and the IP at the network layer. For the purpose of real-time transmission of the OPC UA PubSub data stream, form (2) is used to map the NetworkMessage message to the data. At the link layer, since it bypasses the transport layer and the network layer, our mapping method will provide better real-time guarantee. In completing NetworkMessage mapping, the TSN stream identifier is added to the header according to the IEEE802.1 standard.

**Figure 4:**
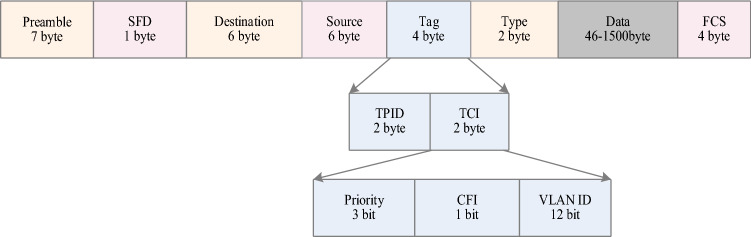
TSN header package.

As shown in Fig. 4, flow identification involves VLAN ID as the destination of MAC, Submitted to PublishconnectionType for system configuration. The specific Publishconnection object parameters include PublisherID, TransportProfileUri, Address, and ConnectionProperties. Before completing the NetworkMessage mapping, the created DataSet, DataSetMessage, and NetworkMessage configurations are set according to the OPC UA PubSub protocol. I will not repeat them here.

**Figure 5:**
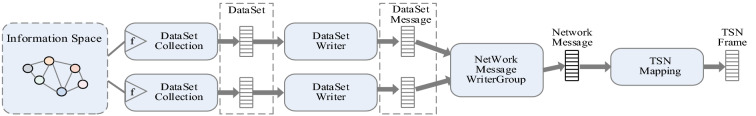
NetworkMessage mapping process.

As shown in Fig. 5, first, we collect the information obtained from the address space into the DataSet, and secondly, map it to the DataSetMessage through the DataSetWriter, then one or more groups of DataSetMessage are mapped to the NetworkMessage through the NetWorkMessage WriterGroup, and finally, the NetworkMessage gets the TSN Frame through the TSN Mapping.

### System model

In this paper, we use application diagrams and structural diagrams to model TT traffic and network topology. An application diagram $G_{p} (T_{p} ,F_{TT} )$ represents the flow graph. The function $T_{p}$ represents the directed graph's vertices and performs the scheduling calculation task for *TT* traffic; $F_{TT}$ represents the edges of the directed graph, which is composed of TT traffic. The structure diagram is represented by an undirected graph $G({\text{V}},{\text{E}})$, where *V* represents a node of an undirected graph composed of a host and a switch and *E* represents a collection of full-duplex physical links between adjacent nodes.

We map the application diagram to the architecture diagram, and the result is the scheduling process for TT traffic. The process is as follows: first, the scheduled calculation task is assigned to the source node. Then, TT traffic that satisfies the scheduling calculation result is mapped to the full-duplex physical link. Finally, it is transmitted to the destination node according to the routing path. We assume that all hosts and TSN switches have achieved IEEE802.1 AS global time synchronization in the mathematical model.

In the TSN network, the switch, and the host exchange three kinds of traffic: TT traffic, AVB traffic, and BE traffic. To ensure interference-free transmission of TT traffic, we use the scheduling algorithm to generate a Gate Control List. The GCL only contains the static schedule of TT traffic, and the transmission of AVB traffic and BE traffic is performed after the transmission of TT traffic is completed.

The time-sensitive data stream is sent from the source node to the destination node. The time-sensitive data stream is represented by the set *s*, $s = \{ s_{1} ,s_{2} ,s_{3} ,...,s_{n} \}$. We use $s^{TT}$ to represent the TT traffic set, with the quadruple $s_{i}$ to represent TT traffic, where $s_{i} = \{ f_{route} ,f_{size} ,f_{period} ,f_{deadline} \}$. The function $f_{route}$ represents the routing link set of TT traffic, where $f_{route} = \{ f_{sender} ,...,f_{receiver} \}$. The TT traffic transmitted in the link contains at least one TSN frame, and $f_{period}$ represents the transmission period of TT traffic; $f_{size}$ represents the load size of each TSN frame multiplied by the number of TSN frames in a transmission period; $f_{deadline}$ represents the maximum allowable end-to-end delay. According to the assumption of^31,32^, TT traffic is only kept in TSN within the processing time of the switch.

To improve the algorithm's applications, we consider the impact on the cost function $\cos t(s)$ from three aspects of data flow: scheduling performance, delayed performance, and routing hop performance, which are represented by the functions $c_{1} (s)$, $c_{{2}} (s)$, and $c_{{3}} (s)$, respectively. The mathematical model of OPC UA Over TSN data flow routing and scheduling is established, as shown in Eq. (1).

1 $$\cos t(s) = w_{1} \times c_{1} (s){ + }w_{{2}} \times c_{{2}} (s){ + }w_{{3}} \times c_{{3}} (s)$$

In the formula, $w_{1}$,$w_{{2}}$, and $w_{{3}}$ are the weights of the influencing factors, and the sum is as shown in Eq. (2).

2 $$w_{1} + w_{2} + w_{3} = 1$$

The first objective function $c_{1} (s)$ represents the level of ability to schedule NetworkMessage data, as shown in Eq. (3), where $s^{TT}$ represents TT traffic waiting to be scheduled. When a NetworkMessage is schedulable, $f_{{s_{i} }} = 0$, otherwise $f_{{s_{i} }} = 1$.

3 $$c_{1} (s) = \sum\limits_{{s_{i} \in s^{TT} }} {f_{{s_{i} }} }$$

There are *n* data flows in the link, where the scheduled situation is represented by the set of $F_{s} = \{ f_{{s_{1} }} ,f_{{s_{2} }} ,...,f_{{s_{n} }} |1 \le i \le n,s_{i} \in s^{TT} \}$.

The delay function $c_{{2}} (s)$ is the sum function of end-to-end delay. End-to-end delay is shown in Eqs. (4) and (5), where $t_{propagation}$ is the propagation delay, $t_{TimeDelay}$ is the transmission delay, and $t_{Queue}$ is the queuing delay. The variables $a_{1}$, $a_{2}$, and $a_{3}$ represent the weights of the two types of delay on the overall delay, and their sum is 1.

4 $$t_{e2eDelay} = a_{1} \times t_{propagation} + a_{2} \times t_{TimeDelay} + a_{3} \times t_{Queue}$$

5 $$a_{1} + a_{2} + a_{3} = 1$$

The propagation delay calculation formula in the process of NetworkMessage transmission is shown in Eq. (6).

6 $$t_{propagation} = f_{size} /v_{c}$$

The $v_{c}$ represents the speed of electromagnetic wave transmission. To ensure the deterministic transmission of TT traffic, the switch nodes in the Gate Control List are activated simultaneously in the network topology, in a fixed cycle.

TT traffic is routed according to the routing table $f_{route}$ and sent from one node to another. During transmission, the data stream is occupied exclusively by the time slot of the routing link. In the channel, the calculation formula for the transmission delay of TT traffic is shown as Eq. (7), where *bw* represents the bandwidth of the corresponding link.

7 $$t_{TimeDelay} = f_{size} /bw$$

In the TSN switch port, we set up a queue for TT traffic scheduling. To ensure the determinism of scheduling, we set a guard band for TT traffic^31,32^. During $t_{TimeDelay}$ and $t_{propagation}$, the link is exclusive.

The queuing delay of TT traffic in the transmission process is shown in Eq. (8), where c is a constant.

8 $$t_{queue} = \frac{{t_{TimeDelay}^{{ \, \frac{1}{{2(1 - {\text{c}})}}}} }}{{{\text{f}}_{{{\text{size}}}} \times \left( {1 - t_{TimeDelay}^{{ \, \frac{1}{{2(1 - {\text{c}})}}}} } \right)^{{{\text{c}}/(1 - {\text{c}})}} }}$$

When TT traffic is received by the next-hop node, the scheduling calculation task is executed. According to the propagation delay $t_{propagation}$ and transmission delay $t_{TimeDelay}$ between the links, the overall delay $c_{{2}} (s)$ from the source node to the destination node is the function shown in Eq. (9).

9 $${\text{c}}_{2} (s) = \sum\limits_{{r_{ij} \in f_{route} }} {r_{ij} \times t_{e2eDelay} }$$

We assume that the network topology contains *N* nodes, and the link occupancy situation can be represented by an *N* matrix $f_{route}$ containing $r_{ij}$, where the value range of *i* and *j* is $(1 \le i,j \le N)$, and TT traffic arrives at node *j* via node *i*, $r_{ij} = 1$ otherwise $r_{ij} = 0$, as shown in Eq. (10).

10 $$f_{route} { = }\left( {\begin{array}{*{20}c} {r_{11} } & {r_{12} } & {r_{13} } & {...} & {r_{1N} } \\ {r_{21} } & {r_{22} } & {r_{23} } & {...} & {r_{2N} } \\ {r_{31} } & {r_{32} } & {r_{33} } & {...} & {r_{3N} } \\ {...} & {...} & {...} & {...} & {...} \\ {r_{N1} } & {r_{N2} } & {r_{N3} } & {...} & {r_{NN} } \\ \end{array} } \right)$$

The third objective function $c_{{3}} (s)$ represents the routing hop of NetworkMessage data from the source node to the destination node, as shown in Eq. (11), where $e_{ij}$ represent weight.

11 $$c_{{3}} (s) = \sum\limits_{i,j \in N} {r_{ij} } \times e_{ij}$$

To ensure the determinism of TT traffic transmission, we need to restrict the network accordingly. This paper aims to provide algorithms for minimizing delay of TT traffic routing and scheduling under specified conditions. The core of the routing problem is to reduce the search space of TT traffic and obtain a set of feasible paths. The core of the scheduling problem is to determine the transmission time slot of TT traffic through an algorithm, to optimize the rate of scheduling success.

**Condition 1** The sending time of TT traffic should be greater than or equal to 0.

$$t_{StartTime} \ge 0$$

**Condition 2** The link should be monopolized during the transmission of TT traffic.

**Condition 3** The TSN switch should not buffer TT traffic but only store and forward TT traffic^31,32^. The sending time of TT traffic to the next-hop node should be greater than or equal to the corresponding receiving time.

$$t_{NextStartTime} \ge t_{StartTime} + t_{transmission} + t_{TimeDelay}$$

**Condition 4** The transmission of TT traffic should be carried out in the path order specified by the route.

$$t_{StartTime}^{i} < t_{StartTime}^{j} (i < j)$$

**Condition 5** The TT traffic that executes the scheduling calculation task must be sent to the next node within the deadline.

$$t_{StartTime} + t_{{{\text{propagation}}}} + t_{TimeDelay} \le f_{deadline}$$

## CKSPACO algorithm

Our CKSPACO algorithm contains three sub-algorithms: the cluster analysis algorithm, the K-Shortest-Paths algorithm, and the improved ant colony algorithm.

### K-means cluster algorithm

**Figure 6:**
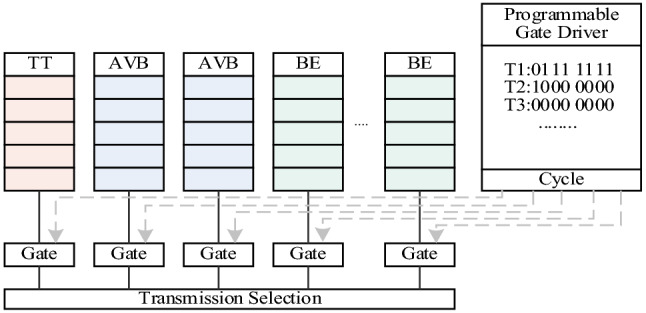
Working principle of IEEE802.1Qbv time-aware-shaper shaper.

A time-aware shaper (TAS) is defined in *IEEE802.1 Qbv*, as shown in Fig. 6. It contains time-aware queues and gate control lists. The data flow enters different queues according to category, and the Gate Control List handles the opening and closing of the gate.

In the configuration of the TAS, we set up a single transmission queue for TT traffic. Considering that the network data set contains multiple traffic types (TT, AVB, BE) and that our algorithm only considers the routing and scheduling of TT traffic, the data set needs to be classified. We obtained the TT traffic data source through a clustering algorithm. According to the Gate Control List, we can schedule the data stream periodically to ensure interference-free transmission and provide deterministic end-to-end delay.

The frame format used in the encoding of NetworkMessage information at the data link layer is IEEE802.1Q^33^. The TCI of the tag contains a 12-bit VLAN Identifier (VID) to distinguish frames of distinct traffic. Three-bit priority is used to indicate the QoS priority of the frame and determine the type of traffic. We map the three types of traffic to different priorities. TT traffic is indicated by 111, 110, and 101 indicate AVB traffic, and 100, 011, 010, 001, and 000 indicate BE traffic.

In the process of information processing, unsupervised learning methods such as anomaly detection^34^, dimensionality reduction^35^, and clustering^36^ are often used. Considering that TT traffic needs to be classified in three categories of data streams, we adopt a clustering method. In the K-Means clustering algorithm, we classify the data stream by identifying the priority of the frame format in the OPC UA PubSub NetworkMessage.

The core of the clustering algorithm involves feature extraction, similarity calculation, and grouping^37^. We randomly select three initial cluster centers $c_{i} \, (1 \le i \le 3)$ from the network data and determine the distance between each time-sensitive data stream in the data set and the Euclidean distance to the center of the cluster^38,39^. Then we target the nearest cluster center $c_{i}$ of the data object, and assign the time-sensitive stream of the corresponding category to it, calculating the average value of the data in each cluster as the new cluster center proceeds to the next iteration, until the cluster center no longer changes or reaches the maximum number of iterations^40,41^. Finally, three different types of time-sensitive data streams are produced.

Through the clustering algorithm, we can determine the quantity of time-triggered traffic from complex NetworkMessages and use it as the data source in the subsequent routing and scheduling algorithms. Effectively reduce the time complexity of the overall algorithm. The pseudo-code of Procedure One is as follows.
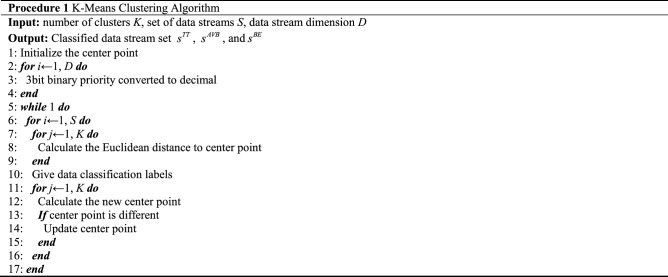


### K-shortest paths algorithm

Architecture diagram *G (V, E)* represents the TSN network topology, where *V* is a collection of *N* nodes, including switch *R* and host *H*, $V = R \cup H$, *E* is a collection of *m* links, $E = \{ e_{1} ,...,e_{m} |m \ge 1\}$, and the weights from node *i* to node *j* in the set can be represented by $e_{{{\text{ij}}}}$. The weights between adjacent nodes in the network topology can be represented by matrix *E*, as shown in Eq. (12).

12 $$E = \left( {\begin{array}{*{20}c} {e_{11} } & {{\text{e}}_{12} } & {e_{13} } & {...} & {e_{1N} } \\ {e_{21} } & {e_{22} } & {e_{23} } & {...} & {e_{2N} } \\ {e_{31} } & {e_{32} } & {e_{33} } & {...} & {e_{3N} } \\ {...} & {...} & {...} & {...} & {...} \\ {e_{N1} } & {e_{N2} } & {e_{N3} } & {...} & {e_{NN} } \\ \end{array} } \right)$$

In the process of TT traffic transmission, routing is the receiving and forwarding aspect. When TT traffic is routed through a TSN switch, we try to limit the number of TT traffic routing hops to less than eight hops. Considering that the data stream path finding process increases in complexity with the growth of the network topology, we use the KSP algorithm to optimize network routing^42^. This algorithm can determine the first *k* shortest paths by weight from the source node to the destination node, reduce the search space of TT traffic by generating the shortest path group, and speed up scheduling algorithm search efficiency^43^. The KSP algorithm consists of two parts, recursion; and determining the shortest path. Finally, the result of the algorithm is used as a new routing table to process the scheduling of TT traffic.

According to the recursion concept, we use the deviation path algorithm to find the shortest path $p_{k}$ from the source node to the destination node, where the set of paths $p_{k} = \{ f_{sender} ,...,f_{receiver} \}$. Then we delete the source node and the destination node in the path set $p_{k}$, label the remaining nodes in the set as deviating nodes in the routing order and select the new shortest path in the path set determined for the new nodes. The first *k* shortest paths from the source node to the destination node are derived by iteration $p = \{ p_{1} ,...,p_{k} |k \ge 1\}$. The $f_{route}$ represents the best routing path selected for TT traffic in the path set *p*.

The pseudo-code of procedure two is as follows.
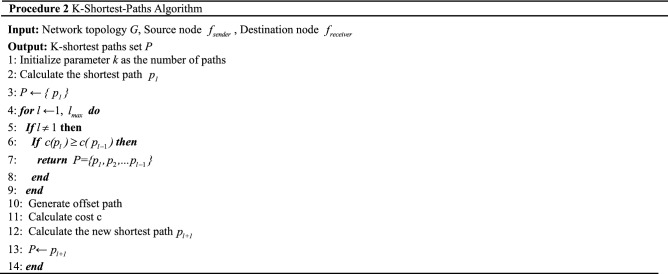


### Improved ant colony algorithm

To ensure that the data stream schedule in the link can be transmitted to the destination node with minimum delay, we use an improved ant colony optimization algorithm to find the minimum delay of the data stream during routing and determine the optimal scheduling plan^44,45^.

To ensure efficiency in the convergence rate of the ant colony algorithm, it is necessary to prevent the ant colony from falling into a local optimum due to a fast convergence rate^46–49^. We introduce the concept of information entropy to express the pros and cons of each path determined by the ant colony. By calculating the information entropy, we make adjustment the parameters to the pheromone heuristic factor α and the expected heuristic factor β. In the optimal ant colony path, when the ant colony selects the next-hop path, if each point's probability is the same, then the information entropy is at the maximum value. The formula is shown in Eq. (13).

13 $$H(x) = - \sum\limits_{t = 1}^{r} {p(x_{t} )\log p(x_{t} )}$$

The set *X* of next-hop path alternatives is represented as ${{X}} = \{{x}_{1}, {{x}}_{2},\ldots,{{x}}_{r}\}$, and P is the probability of corresponding set elements. To normalize the information entropy, we need to find the maximum quantity of information entropy, which is when the probability of the next hop of the path set elements is equal, as shown in Eq. (14).

14 $$H_{\max } = - \sum\limits_{i = 1}^{r} {P_{i} } \log P_{i} = - \sum\limits_{i = 1}^{r} \frac{1}{r} \log \frac{1}{r}$$

The maximum value of information entropy is $H_{\max }$, and r represents the number of elements in the next-hop selection set. Normalization processing is shown in Eq. (15).

15 $$H^{^{\prime}} = H(x)/H_{\max }$$

The value of $\rho$ influences the convergence rate of the ant colony algorithm and the global searchability.

Accordingly, we propose a formula for adjusting ρ in real time based on a negative feedback mechanism, which reduces premature convergence of the positive feedback mechanism that depends on pheromone concentration in the ant colony algorithm, as shown in Eq. (16).

16 $$\rho { = [(}\rho_{\max } { - }\rho_{\min } {)} \times {\text{t + (T - 1)}} \times \rho_{\min } + \rho_{\max } ]/{\text{T}}$$

The maximum evaporation coefficient is $\rho_{\max }$, $\rho_{\min }$ is the minimum evaporation coefficient, t is the current iteration number, and T is the maximum iteration number. The pseudo-code of Procedure Three is as follows.
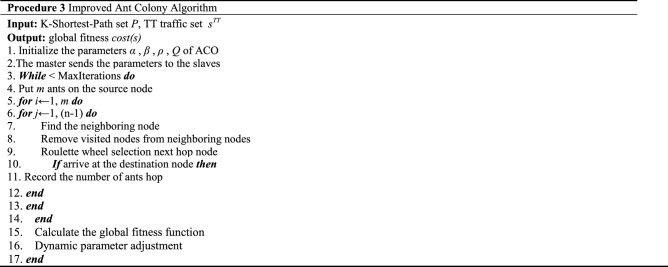


## Simulation result

To prove the superiority of the algorithm, this paper compares the CKSPACO and the IACO^12^ algorithms. The program is written using Matlab, and the hardware specifications are Intel Core i7-8750H, 8 GB RAM. We consider two factors that affect the transmission efficiency of time-triggered traffic: the quantity of time-triggered traffic, and the network topology scale. According to these factors, the improved algorithm was tested in various scenarios. In our mathematical model, the weight parameters are set to the following values: $w_{{_{{1}} }}$ = 0.6, $w_{{2}}$ = 0.2, $w_{{3}}$ = 0.2, $a_{1}$ = 0.2, $a_{2}$ = 0.3, *c* = 0.5.

**Figure 7:**
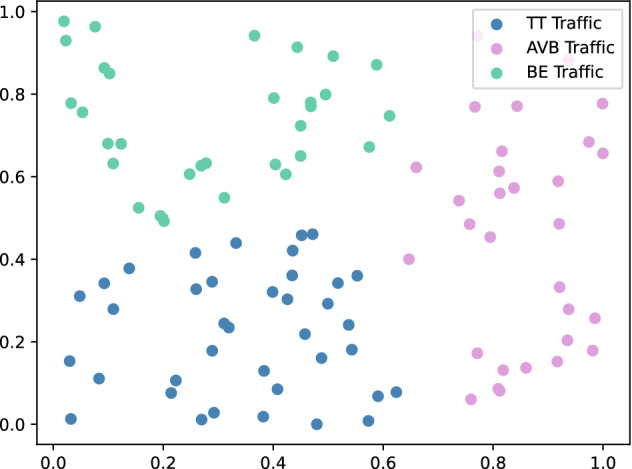
The number of data streams is 100.

We selected 100, 250, and 500 data streams from the network data set as the input of the K-Means clustering algorithm. When the input data stream is 100, the clustering result contains 38 TT traffic, 31 AVB traffic, and 31 BE traffic. The result is shown in Fig. 7.

The other two data stream distributions are shown in Table 1.

**Table 1:** Data stream category result.

Algorithm category	The number of TT traffic		
	38 TT traffic	123 TT traffic	208 TT traffic
KSPACO	1.7	4.8	11.7
IACO	3.0	11.2	23.4

In the KSP algorithm, we set up three scales of network topology scales of 10, 20, and 30. Take the network topology of 20 nodes as an example, and calculate the first *k* shortest paths from the source node 1 to the destination node 20, expressed in a matrix. Generate a new routing table to reduce the search range of the improved ant colony algorithm.

$$\left( {\begin{array}{*{20}c} {1} & {8} & {{15}} & {{14}} & {{20}} & {0} \\ {1} & {3} & {{17}} & {{14}} & {{20}} & {0} \\ {1} & {2} & {4} & {{19}} & {{20}} & {0} \\ {1} & {8} & {9} & {{12}} & {{14}} & {{20}} \\ {1} & {3} & {6} & {{17}} & {{14}} & {{20}} \\ \end{array} } \right)$$

Finally, we use the quantity of TT traffic obtained by the K-Means clustering algorithm as the data source for routing and scheduling in the improved ant colony algorithm. The KSP algorithm utilizes the first *k* shortest paths for the routing table of the improved ant colony algorithm. Using the ant colony algorithm, we obtain the single-hop path, the delay of the global path, and improve TT traffic scheduling performance.

**Figure 8:**
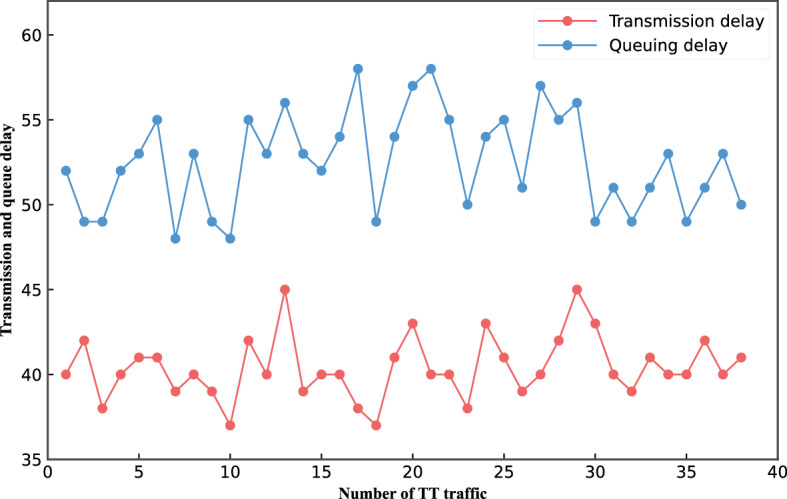
38 TT traffic transmission delay and queuing delay.

Figure 8 shows the transmission delay and queuing delay generated when 38 TT traffic passes through nodes in the network topology. We can know that One TT traffic transmission delay and queuing delay are stable at 40 μs and 50 μs.

**Figure 9:**
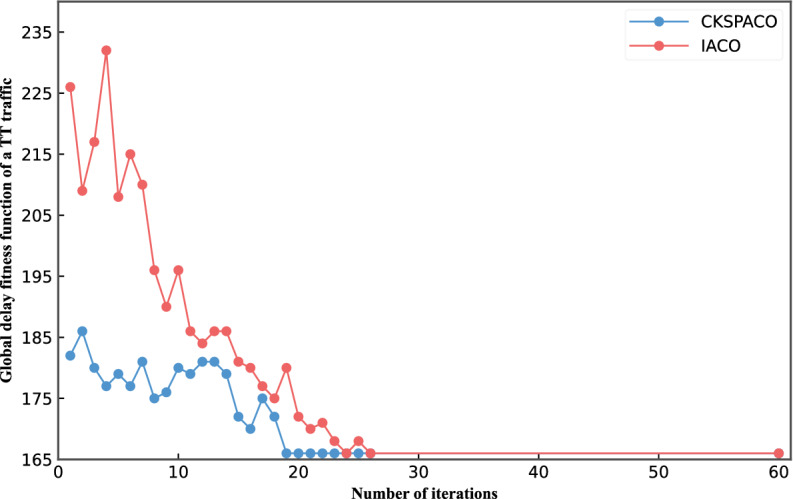
One TT traffic global delay.

Figure 9 shows the CKSPACO and IACO algorithms' global delay function in a TT traffic routing and scheduling process. From the figure, we see that the random search strategy of the IACO algorithm greatly affects the global delay of the algorithm in the beginning. Still, it also converges to the corresponding value as the number of iterations increases. Compared with the IACO algorithm, the CKSPACO algorithm performs better in the convergence rate and the number of iterations.

**Figure 10:**
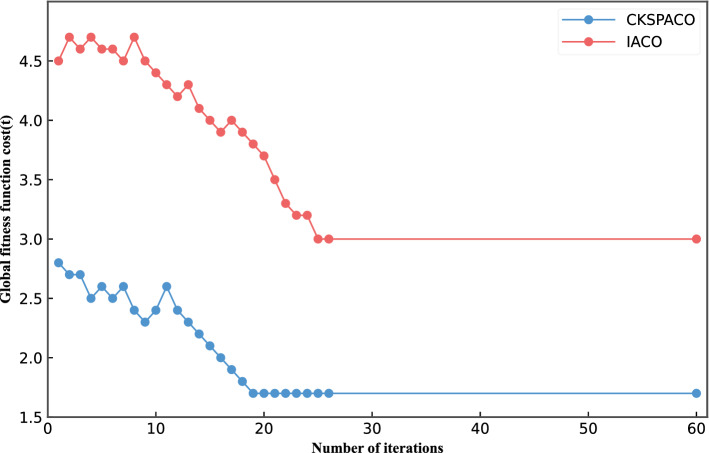
Comparison of overall cost fitness function between CKSPACO algorithm and IACO algorithm.

Figure 10 shows performance in terms of the global fitness function cost of the CKSPACO algorithm and the IACO algorithm. We test the algorithms based on 38 TT traffic with 20 network topology nodes. Because the IACO algorithm schedules TT traffic based on a fixed routing strategy, there is a large queuing delay in the process, which increases overall cost. Compared with the IACO algorithm, the CKSPACO algorithm provides a dynamic routing strategy for TT traffic that ensures a good convergence rate and number of iterations of the algorithm. On the whole, the CKSPACO algorithm can provide better scheduling performance.

We test algorithm performance by adjusting the quantity of TT traffic, the scale of the network topology, and network bandwidth. The quantity of TT traffic in different scenarios is obtained by the clustering algorithm, represented by the set *C*. *C* = {38,123,208}. Set *R* represents different network topology scales, *R* = {10,20,30}; the combined influence of the two factors is used to test the algorithm.

When the network topology scale is 20 nodes, the algorithm performance is tested by adjusting the quantity of TT traffic to be dispatched, as shown in Table 2.

**Table 2:** Data the influence of the quantity of TT traffic on the overall fitness function.

The number of data stream	Data stream category		
	TT traffic	AVB traffic	BE traffic
250	123	84	43
500	208	178	114

As the quantity of TT traffic increases, the fitness functions of the two algorithms gradually become larger. Since the IACO algorithm utilizes a fixed routing strategy, its fitness function value increases significantly as the quantity of TT traffic increases. Although the value of the CKSPACO algorithm fitness function also increases with increase in the quantity of TT traffic, it still maintains good scheduling performance.

When the quantity of TT traffic to be scheduled is 38, algorithm performance is tested by adjusting the network topology scale, as shown in Table 3.

**Table 3:** The influence of network topology scale on the overall fitness function.

Algorithm category	Network topology scale		
	10 topology	20 topology	30 topology
KSPACO	2.1	1.7	1.6
IACO	3.1	3.0	2.8

As the scale of the network topology expands, the values of the fitness functions of the two algorithms fluctuate less, because we limit the number of routing hops to less than 8 hops in the KSP algorithm, and the scale of TT traffic to be scheduled is small. The IACO algorithm based on fixed routing is virtually unaffected by changes in the network topology. In the CKSPACO algorithm, the fitness function value based on 10 network nodes fluctuates slightly, due to the connectivity of our initially generated network topology.

## Conclusion

In OPC UA over TSN, we have solved the routing and scheduling problems of time sensitive data streaming in OPC UA PubSub NetworkMessage with the improved ant colony algorithm CKSPACO, of great significance to the combination of OPC UA with TSN. Using the improved algorithm to schedule TT traffic can effectively increase algorithm convergence rate and improve the real-time performance of network message transmission.
